# DNA Damage and Repair Biomarkers in Cervical Cancer Patients Treated with Neoadjuvant Chemotherapy: An Exploratory Analysis

**DOI:** 10.1371/journal.pone.0149872

**Published:** 2016-03-01

**Authors:** Patrizia Vici, Simonetta Buglioni, Domenico Sergi, Laura Pizzuti, Luigi Di Lauro, Barbara Antoniani, Francesca Sperati, Irene Terrenato, Mariantonia Carosi, Teresa Gamucci, Rosanna Dattilo, Monica Bartucci, Cristina Vincenzoni, Luciano Mariani, Enrico Vizza, Giuseppe Sanguineti, Angiolo Gadducci, Ilio Vitale, Maddalena Barba, Ruggero De Maria, Marcella Mottolese, Marcello Maugeri-Saccà

**Affiliations:** 1 Division of Medical Oncology B, “Regina Elena” National Cancer Institute, Rome, Italy; 2 Department of Pathology, “Regina Elena” National Cancer Institute, Rome, Italy; 3 Biostatistics-Scientific Direction, “Regina Elena” National Cancer Institute, Rome, Italy; 4 Medical Oncology Unit, ASL Frosinone, Frosinone, Italy; 5 Department of Hematology, Oncology and Molecular Medicine, Istituto Superiore di Sanità, Rome, Italy; 6 Drug Discovery Program, Rutgers Cancer Institute of New Jersey, New Brunswick, New Jersey, United States of America; 7 Department of Surgery, Gynecologic Oncology Unit, “Regina Elena” National Cancer Institute, Rome, Italy; 8 HPV-UNIT, “Regina Elena” National Cancer Institute, Rome, Italy; 9 Department of Radiotherapy, “Regina Elena” National Cancer Institute, Rome, Italy; 10 Department of Experimental and Clinical Medicine, Division of Gynecology and Obstetrics, University of Pisa, Pisa, Italy; 11 Department of Biology, University of Rome "Tor Vergata", Rome, Italy; 12 Scientific Direction, “Regina Elena” National Cancer Institute, Rome, Italy; Florida State University, UNITED STATES

## Abstract

Cervical cancer cells commonly harbour a defective G_1_/S checkpoint owing to the interaction of viral oncoproteins with p53 and retinoblastoma protein. The activation of the G_2_/M checkpoint may thus become essential for protecting cancer cells from genotoxic insults, such as chemotherapy. In 52 cervical cancer patients treated with neoadjuvant chemotherapy, we investigated whether the levels of phosphorylated Wee1 (pWee1), a key G_2_/M checkpoint kinase, and γ-H2AX, a marker of DNA double-strand breaks, discriminated between patients with a pathological complete response (pCR) and those with residual disease. We also tested the association between pWee1 and phosphorylated Chk1 (pChk1), a kinase acting upstream Wee1 in the G_2_/M checkpoint pathway. pWee1, γ-H2AX and pChk1 were retrospectively assessed in diagnostic biopsies by immunohistochemistry. The degrees of pWee1 and pChk1 expression were defined using three different classification methods, i.e., staining intensity, Allred score, and a multiplicative score. γ-H2AX was analyzed both as continuous and categorical variable. Irrespective of the classification used, elevated levels of pWee1 and γ-H2AX were significantly associated with a lower rate of pCR. In univariate and multivariate analyses, pWee1 and γ-H2AX were both associated with reduced pCR. Internal validation conducted through a re-sampling without replacement procedure confirmed the robustness of the multivariate model. Finally, we found a significant association between pWee1 and pChk1. The message conveyed by the present analysis is that biomarkers of DNA damage and repair may predict the efficacy of neoadjuvant chemotherapy in cervical cancer. Further studies are warranted to prospectively validate these encouraging findings.

## Introduction

Eukaryotic cells are constantly exposed to endogenous and exogenous sources of DNA damage. The transmission of undamaged DNA to the offspring is ensured by a complex molecular network, the DNA damage response (DDR), which operates through the coordinated activity of cell cycle checkpoints, DNA repair mechanisms and apoptotic pathways [1, 2]. The presence of genetic lesions triggers checkpoint-mediated arrest of the cell cycle [2]. This event enables DNA repair effectors and apoptotic pathways to repair the lesion or eliminate irremediably damaged cells, respectively.

Cancer cells aberrantly use DNA repair mechanisms to survive stressful conditions, such as exposure to chemotherapy [2]. A common trait to a variety of tumors is the defective nature of the G_1_/S-phase checkpoint, stemming from mutational or functional inactivation of p53 or retinoblastoma protein (pRb) [3]. When this occurs, cancer cells become extremely dependent on the G_2_/M checkpoint for cell cycle arrest and DNA repair [3]. The ataxia telangiectasia and Rad3-related protein (ATR)-Checkpoint kinase 1 (Chk1)-Wee1-like protein kinase (Wee1) cascade represents the core of the G_2_/M checkpoint, whose activation leads to the inhibition of the cyclin-dependent kinase 1 and culminates into checkpoint-mediated cell cycle arrest [3]. In such a manner, cancer cells have the time to correct chemotherapy-induced DNA lesions, avoiding entry into a lethal mitosis known as mitotic catastrophe [4]. G_2_/M checkpoint dependency in a p53-defective molecular background is a concept currently exploited for the clinical development of synthetic lethality-based therapeutics. When G_1_/S-phase checkpoint-defective cells are exposed to chemotherapeutics, the concomitant pharmacological inhibition of G_2_/M checkpoint kinases is deleterious for cell fitness [3].

We reasoned that G_2_/M checkpoint “addiction” for compensating p53 or pRb defects upon exposure to genotoxic agents can be exploited in the search for predictive biomarkers foreseeing chemotherapy sensitivity/resistance. In this exploratory analysis we focused on cervical cancer, the prototype of p53- and pRb-defective tumors. Indeed, human papillomavirus E6 and E7 oncoproteins promote ubiquitin-mediated degradation of p53 and pRb, respectively [5]. We thus retrospectively investigated the association between the levels of DNA damage and repair biomarkers, assessed in bioptic samples collected from untreated patients at the time of diagnosis, and pathological complete response (pCR) after neoadjuvant chemotherapy, i.e., chemotherapy delivered in the timeframe between diagnostic biopsy and the surgical resection. All the patients were homogenously treated with paclitaxel, ifosfamide and cisplatin (TIP regimen). We focused on phosphorylated Wee1 (pWee1) as a proxy of G_2_/M checkpoint activation, and phosphorylated H2A Histone Family Member X (γ-H2AX) as a marker of DNA double-strand breaks. Phosphorylated Chk1 (pChk1) was tested in a fraction of samples for a signaling study.

## Materials and Methods

### Study Participants and Procedures

Fifty-two histologically confirmed cervical cancer patients (stage Ib2-IIIa) who received neoadjuvant chemotherapy were included in this retrospective analysis. All patients were treated with the TIP regimen (paclitaxel 175 mg/m^2^ on day 1 + ifosfamide 2500 mg/m^2^ on days 1 and 2 + cisplatin 50 mg/m^2^ on day 2 every 21 days for three or four cycles) followed by radical surgery. Patients were considered eligible if they completed the planned treatment, data on clinical features and treatment outcomes were available, and the amount of biological materials in their biopsies was sufficient for molecular analyses. pCR was defined as no residual disease in surgical samples. The immunohistochemical assessment of pWee1, γ-H2AX, and pChk1 was performed in formalin-fixed paraffin-embedded (FFPE) tissues, obtained from the biological specimens collected through bioptic procedures in untreated patients, using the following antibodies: anti-phospho-H2AX (Ser139) (clone JBW301) mouse monoclonal antibody (MAb) (Upstate, NY, USA) at the dilution of 1:500, anti-phospho-Wee1 (Ser642) (clone D47G5) rabbit MAb (Cell Signaling, Danvers, MA, USA) at the dilution of 1:100, and anti-phospho-Chk1 (Ser345) (clone 133D3) rabbit MAb (Cell Signaling, Danvers, MA, USA) at the dilution of 1:100. Immunohistochemical staining was performed in an automated autostainer (BOND-III, Leica, Milan, Italy) by a biotin-free polymeric horseradish peroxidase (HRP)-linker antibody conjugate system (Leica, Milan, Italy). For each tumor, three different, 3 μm paraffin sections were analyzed and examined by light microscopy. Immunoreaction of tumor cells was counted in four high-power fields (400x magnification) per section. pWee1 and pChk1 were considered positive when ≥10% of the neoplastic cells showed a distinct nuclear immunoreactivity. pWee1 and pChk1 were graded on a four-grade scale based on staining intensity (0: negative, 1+: weak, 2+: moderate, 3+: strong). Tumors were classified as negative (0 = pWee1^neg^ and pChk1^neg^) or positive (1–3 = pWee1^pos^ and pChk1^pos^).The Allred scores were obtained as previously described [6], considering staining intensity and percentage of tumor-expressing cells, and reported according to a scale of 0 to 8. Tumors were classified as low expressing if the Allred score was ≤ 2 (pWee1^allred low^, pChk1^allred low^), or as high expressing if the Allred score was > 2 (pWee1^allred high^, pChk1^allred high^). The multiplicative scores were obtained by multiplying staining intensity x the percentage of tumor-expressing cells, and were expressed on a scale of 0 to 300. Tumors were classified as low expressing (pWee1^multi low^ and pChk1^multi low^) or high expressing (pWee1^multi high^ and pChk1^multi high^) using the median score of all tumors as a cut-off point. γ-H2AX expression was considered as the percentage of tumor-expressing cells and analyzed both as continuous (γ-H2AX^cont^) and as categorical variable, whose modality was defined using the median score of all tumors (γ-H2AX ^low^ and γ-H2AX ^high^). Tumor samples were evaluated independently by two investigators (SB and MC) who were blinded to treatment outcomes, and discordant cases were reviewed (MM). This retrospective study was conducted in accordance with the Declaration of Helsinki and was approved by the Ethic Committee of “Regina Elena” National Cancer Institute of Rome, the coordinating centre. Written informed consents were secured before chemotherapy.

### Statistical analysis

Cancer- and patient-related features were descriptively characterized for all the patients included in the present analysis. Medians and ranges were used to report on continuous variables, while categorical variables were expressed by frequencies and percentage values. In order to assess the relationships between categorical variables the Pearson’s Chi-squared test of independence (2-tailed) and the Fisher Exact test were employed. The use of univariate logistic regression models helped identify variables potentially impacting treatment outcome. Multivariate logistic regression models were built by including variables testing significant at the univariate assessment or identified based on the clinical plausibility of their role in influencing pCR. To estimate the risk of an overfitted multivariate model and examine its stability, an internal validation was carried out using a re-sampling procedure without replacement. To this end, one hundred datasets were generated by randomly removing approximately 20% of the original sample. For each simulation, we repeated the multivariate logistic regression model and the Cohen's Kappa coefficient, the Positive Predictive Value (PPV), the Negative Predictive Value (NPV), Sensibility and Specificity were calculated. We considered statistically significant p values less than 0.05. Statistical analyses were carried out using SPSS software (SPSS version 21, SPSS Inc., Chicago, IL, USA).

## Results

**Fig 1:**
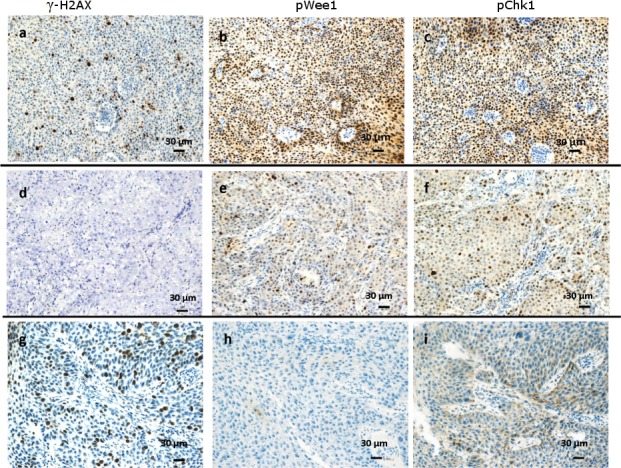
Representative examples of immunohistochemical expression of DNA damage and repair biomarkers in cervical cancer patients. Three consecutive sections for each tumor are showed. (A-C) A triple positive tumor with nuclear γ-H2AX(A), pWee1(B) and pChk1 (C) immunoreactivity.(D-F) A tumor that did not express γ-H2AX (D), and that co-expressed pWee1(E) and pChk1 (F). (G-I) A tumor expressing nuclear γ-H2AX (G) that lacked both pWee1(H) and pChk1 (I) expression.

**Fig 2:**
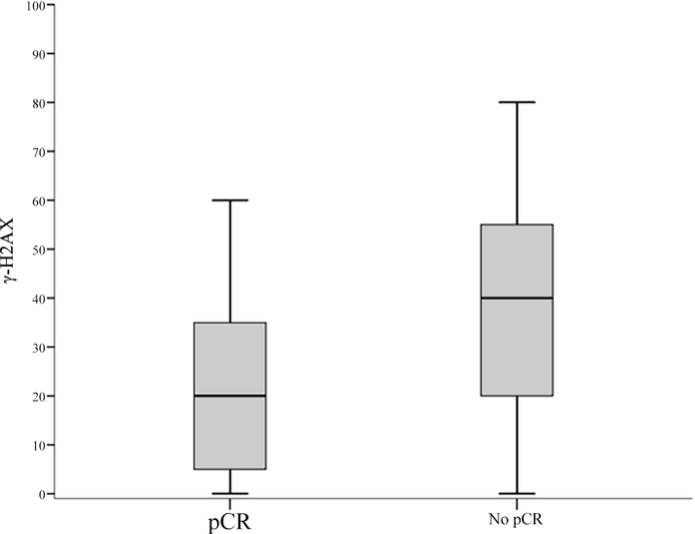
Box plot of the distribution of γ-H2AX values by pathologic complete response. In the figure: the upper horizontal line of the box is the 75th percentile; the lower horizontal line of the box is the 25th percentile; the horizontal bar within box is the median value; the upper horizontal bar outside the box is the maximum value; the lower horizontal bar outside the box is the minimum values.

**Fig 3:**
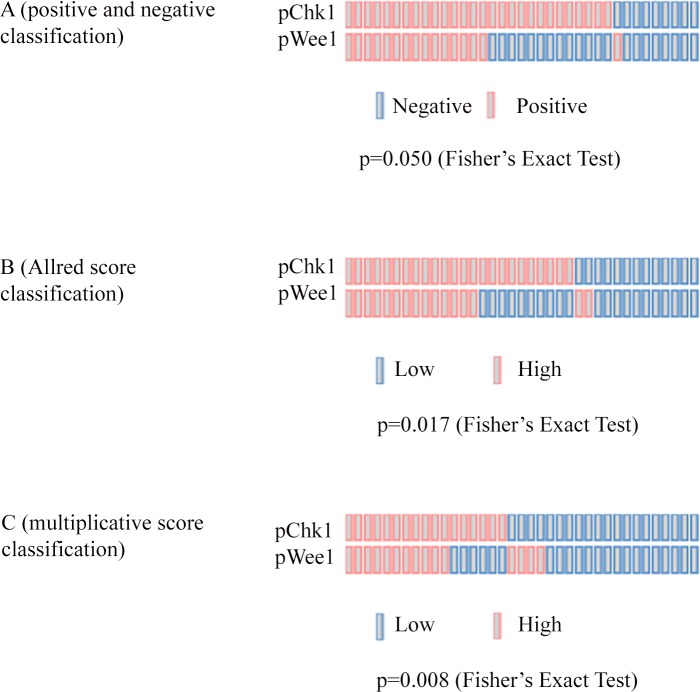
OncoPrints showing the association between pWee1 and pChk1 in 37 cervical cancer samples. (A) Association according to staining intensity-based classification (positive vs negative). (B) Association according to Allred score classification (high vs low). (C) Association according to a multiplicative score classification (high vs low).

Baseline characteristics of the 52 patients included in this study are summarized in Table 1. Median time between diagnostic biopsy and radical surgery was 4.99 months [IQ Range: 4.01–5.83]. All the pre-chemotherapy samples, consisting in diagnostic biopsies, were examined for pWee1 and γ-H2AX, whereas pChk1 data were available for 37 samples. Median percentages of nuclear-expressing cells for pWee1, pChk1 and γ-H2AX were 40% (min/max 10/80), 30% (min/max 10/80) and 30% (min/max 0/80), respectively. Representative immunohistochemical staining patterns are illustrated in Fig 1. As shown in Table 2, we found a statistically significant association between elevated nuclear pWee1 expression and reduced pCR rate. The association tested significant for all the scoring methods investigated (pWee1^pos^ vs pWee1^neg^, p = 0.016; pWee1^allred high^ vs pWee1^allred low^, p = 0.016; pWee1^multi high^ vs pWee1^multi low^, p = 0.034) (Table 2). Likewise, elevated nuclear levels of γ-H2AX were associated with reduced pCR rate, both when considered as categorical and continuous variable (p = 0.037 and p = 0.026 in Table 2 and Fig 2, respectively). When considering the combination of the two markers, only 1 patient out of 16 with double positive tumors experienced a pCR, 9 out of 16 patients with double negative tumors achieve a pCR, and an intermediate outcome was seen in patients whose tumors expressed only one biomarker (p = 0.009) (Table 3). Six out of 8 deaths were observed in double positive tumors (p = 0.013) (Table 3). In the univariate logistic regression model, pWee1 and γ-H2AX were directly associated with pCR (pWee1^pos^ vs pWee1^neg^: Odds Ratio (OR) 5.31, 95% Confidence Interval (CI): 1.42–19.87, p = 0.013; γ-H2AX^high^ vs γ-H2AX^low^: OR 4.20, 95%CI:1.13–15.59, p = 0.032, respectively) (Table 4); the multivariate model confirmed the predictive role of pWee1 and γ-H2AX (Table 4). The internal validation performed through a re-sampling procedure confirmed the robustness of the multivariate model. Concordance, Positive Predictive Value, Negative Predictive Value, Sensitivity and Specificity are shown in Table 5. Finally, when investigating co-expression patterns, we did not observe any association between pWee1 and γ-H2AX (data available upon request), whereas a statistically significant association was reported between pWee1 and pChk1 (Fig 3).

**Table 1:** Baseline characteristics and treatment outcome of cervical cancer patients treated with neoadjuvant chemotherapy (N = 52).

Characteristics		N (%)
**Age at diagnosis** Median (range)		45.5 (37.2–56.0)
**Stage**		
	I	17 (32.7)
	II-III	35 (67.3)
**Histology**		
	Squamous cell carcinoma	43 (82.7)
	Adenocarcinoma	9 (17.3)
**Number of chemotherapy cycles**		
	3	28 (53.8)
	4	24 (46.2)
**Pathological complete response**		
	Yes	16 (30.8)
	No	36 (69.2)

**Table 2:** Association between biomarkers of DNA damage and repair (pWee1 and γ-H2AX) and pathological complete response in cervical cancer patients treated with neoadjuvant chemotherapy (N = 52).

	Pathological complete response		
	No	Yes	Fisher's Exact Test
Biomarker	N (%)	N (%)	P-value
pWee1^neg^	13 (52.0)	12 (48.0)	0.016
pWee1^pos^	23 (85.2)	4 (14.8)	
pWee1^allred low^	13 (52.0)	12 (48.0)	0.016
pWee1^allred high^	23 (85.2)	4 (14.8)	
pWee1^multi low^	14 (53.8)	12 (46.2)	0.034
pWee1^multi high^	22 (84.6)	4 (15.4)	
γ-H2AX^cat low^	15 (55.6)	12 (44.4)	0.037
γ-H2AX^cat high^	21 (84.0)	4 (16.0)	

pWee1, phosphorylated Wee1-like protein kinase; γ-H2AX, phosphorylated H2A Histone Family Member X

**Table 3:** Association between the co-expression of pWee1 and γ-H2AX and A) Pathological complete response (N = 52), B) Death (N = 8).

	**Pathological complete response**		
**A) N = 52**	**No**	**Yes**	**Chi2**
	**N (%)**	**N (%)**	**P-value**
**pWee1**^**neg**^ **/ γ-H2AX**^**cat low**^	7 (43.8)	9 (56.3)	0.009
**pWee1**^**neg**^ **/γ-H2AX**^**cat high**^ **or pWee1**^**pos**^ **/γ-H2AX**^**cat low**^	14 (70.0)	6 (30.0)	
**pWee1**^**pos**^ **/γ-H2AX**^**cat high**^	15 (93.8)	1 (6.2)	
	**Death**		
**B) N = 8**	**No**	**Yes**	**Chi2**
	**N (%)**	**N (%)**	**P-value**
**pWee1**^**neg**^ **/γ-H2AX**^**cat low**^	15 (93.8)	1 (6.2)	0.013
**pWee1**^**neg**^ **/γ-H2AX**^**cat high**^ **or pWee1**^**pos**^ **/γ-H2AX**^**cat low**^	19 (95.0)	1 (5.0)	
**pWee1**^**pos**^ **/γ-H2AX**^**cat high**^	10 (62.5)	6 (37.5)	

pWee1, phosphorylated Wee1-like protein kinase; γ-H2AX, phosphorylated H2A Histone Family Member X

**Table 4:** Uni and multivariate logistic regression models of patient- and disease-related features and pathological complete response.

		Univariate logistic regression model		Multivariate logistic regression model*	
		OR (95%CI)	P-value	OR (95%CI)	P-value
**Variables**					
**Age**	**>45.5 vs ≤45.5**	0.70 (0.21–2.28)	0.549		
**Stage**	**II-III vs I**	1.36 (0.40–4.69)	0.623		
**Histology**	**AC vs SCC**	Not applicable		Not applicable	
**CT cycles**	**4 vs 3**	2.46 (0.71–8.52)	0.156		
**γ-H2AX**	**high vs low**	4.20 (1.13–15.59)	0.032	7.14 (1.30–39.29)	0.024
**pWee1**	**pos vs neg**	5.31 (1.42–19.87)	0.013	8.92 (1.68–47.26)	0.010

* Adjusted for age, stage, number of chemotherapy cycles.

AC, Adenocarcinoma; SCC: Squamous cell carcinoma; CT, Chemotherapy.

**Table 5:** Replication stability of the multivariate analysis after internal validation with a re-sampling procedure. One hundred less-powered simulation datasets were generated, each approximately 80% of the original size.

	Mean	Median	Minimum	Maximum	Standard Deviation
**Cohen's kappa coefficient**	0.575	0.581	0.408	0.715	0.06
**Positive predictive value (PPV)**	0.741	0.742	0.600	0.888	0.05
**Negative predictive value (NPV)**	0.858	0.857	0.800	0.933	0.02
**Sensitivity**	0.660	0.667	0.440	0.800	0.06
**Specificity**	0.898	0.899	0.833	0.966	0.03

## Discussion

In the present study we retrospectively explored the predictive significance of pWee1 and γ-H2AX expression, evaluated in diagnostic biopsies related 52 cervical cancer patients who received neoadjuvant chemotherapy. We also investigated the association between pWee1 and pChk1 in order to provide clues on whether Wee1 activation in cervical cancer is mediated by Chk1. To our knowledge, this is the first study reporting on DNA damage and repair biomarkers in cervical cancer that exploited the concept of the defective nature of the G_1_/S-phase checkpoint. Overall, we observed a statistically significant association between elevated expression of pWee1 and γ-H2AX and reduced rate of pCR. Thus, we provided first hints that the elevated expression of DDR biomarkers in diagnostic samples might be associated with suboptimal efficacy of chemotherapy, evaluated through pCR in surgically resected tumors. We also observed a positive association between pWee1 and pChk1 expression that suggests effective G_2_/M checkpoint activation. We are aware that our results are hypothesis-generating in nature given the retrospective design of the study. Nevertheless, beyond the straightforward analytical approach, our study has some important strengths.

First, the neoadjuvant setting offers multiple advantages for the identification and development of cancer biomarkers: i) the analysis of potential markers in a molecular background not “polluted” by the exposure of previous anticancer treatments, ii) the identification of predictive markers to select patients who will more likely benefit from chemotherapy, iii) the identification of biomarkers that also hold prognostic significance, even though evidence on the association between pCR and long-term survival outcomes in cervical cancer is not as robust as it is in breast cancer [7, 8].

Second, thus far, in cervical cancer the search for predictive biomarkers linked to the increased ability of cancer cells to protect their genome when challenged with chemotherapy or radiotherapy has been exclusively focused on nucleotide excision repair (NER) proteins, and in particular on the excision repair cross-complementation group1 (ERCC1) protein [9–13]. NER is deputed to correct bulky helix-distorting lesions, such as those inflicted on the DNA by platinum-based therapy. However, within the context of the DDR, NER is one of the many distal effectors assigned to maintain genome integrity. A number of molecular networks safeguard the genome, albeit their engagement depends on the type of lesion. DNA repair pathways also include base excision repair (BER), mismatch repair (MMR), direct repair, and the double-strand break (DSB) recombinational repair. This latter encompasses the error-free homologous recombination repair (HRR) and the error-prone non-homologous end-joining (NHEJ) [1]. Therefore, the level of biologic complexity of the DDR might be underestimated when exclusively considering one, or few, components collocated in a specific repair network. Moreover, concerns were raised on the reliability, and biological significance, of ERCC1 detection methods [14, 15]. Conversely, our study focused on master DDR components, whose activation is known to be particularly efficient in cervical cancer.

Next, we hypothesized that endogenous levels of DNA damage, mirrored by γ-H2AX, should have been paralleled by increased expression of pWee1 and pChk1.Even though the ATR-Chk1-Wee1 axis is primarily activated by stretched of single-stranded DNAs, these abnormal structures may generate DSBs upon replication fork collapse [16]. Moreover, we reasoned that the activation of the G_2_/M checkpoint should be particularly proficient in the presence of high basal levels of endogenous DNA damages, representing an adaptive mechanism through which cancer cells counteract oncogene-induced replication stress [16]. Indeed, it is known that ATR and Chk1 suppress the apoptotic response following DNA replication stress [17], and that tumors characterized by elevated levels of replicative stress, such as Myc-driven cancers, are extremely vulnerable to the pharmacological targeting of G_2_/M checkpoint kinases [18–23]. We did not observe any association between pWee1 and γ-H2AX, but rather these endpoints were independently associated with pCR. We can speculate that two independent repair avenues, particularly efficient in cervical cancer, were captured in this study. A suitable candidate is the Ataxia-telangiectasia mutated (ATM)-Checkpoint kinase 2 (Chk2) pathway, which is mainly activated by DSBs [16]. An extensive cooperation exists between the ATM-Chk2 pathway and ATR-Chk1-Wee1 signaling, and ATM also phosphorylates H2AX [16].

Another aspect emerging from this study relates to the association between pWee1 and pChk1 expression. Wee1 is placed downstream Chk1 [3], and Wee1 phosphorylation at Ser642 increases its stability in the nucleus and promotes cell cycle arrest at the G_2_/M transition [24, 25]. However, to our knowledge formal proof that this regulatory mechanism, namely Chk1-driven phosphorylation of Wee1 at Ser642, operates in mammalian cells is still lacking. Current evidence mostly stems from studies using *Xenopus* extracts and *Schizosaccharomyces pombe* as model systems [26, 27]. Even though our study was not designed to generate mechanistic insights into the dynamics governing Wee1 activation, its results provide a suggestion for future preclinical investigations.

A final point that deserves consideration refers to the protective role of G_2_/M checkpoint activation in the context of cancer stem cells [28]. Activation of the axis has been associated with therapeutic resistance in different cancer stem cell models, including brain, lung and colon cancers [29–31]. Multiplying the efforts for establishing a collection of patient-derived cervical cancer stem cells for comprehensive molecular characterization is a strategy that should be pursued to further dissect the relationship existing between G_2_/M checkpoint activation and chemoresistant features. The relevance of this approach is even more evident when considering the need for more accurate cellular and animal models in light of the number of Chk1 and Wee1 inhibitors that entered clinical development [3, 32]. For instance, a phase I trial with the first-in-class Wee1 inhibitor AZD1775 (MK1775) in association with cisplatin and radiation therapy is ongoing (ClinicalTrials.gov Identifier: NCT01958658), and a phase I/II trial in combination with topotecan/cisplatin results as completed (ClinicalTrials.gov Identifier:NCT01076400). Moreover, a phase I-II trial of AZD1775 in combination with chemotherapy has been initiated in patients with TP53-mutated epithelial ovarian, fallopian tube, or primary peritoneal cancer [33].

## Conclusions

To sum up, pWee1 andγ-H2AX expression in pre-chemotherapy samples showed ability to foresee pCR in cervical cancer patients treated with neoadjuvant paclitaxel, ifosfamide and cisplatin. Based on the extremely promising results herein presented prospective validation or, alternatively, ancillary molecular analyses in the context of prospective trials is warranted to better characterize the predictive ability of these biomarkers.

## Abbreviations

ATM: Ataxia-telangiectasia mutated
ATR: ataxia telangiectasia and Rad3-related protein
Chk1: Checkpoint kinase 1
Chk2: Checkpoint kinase 2
DDR: DNA damage response
DSB: double-strand break
pCR: pathological complete response
pChk1: hosphorylated Chk1
γ-H2AX: phosphorylated H2A Histone Family Member X
pRb: retinoblastoma protein
pWee1: phosphorylated Wee1
Wee1: Wee1-like protein kinase
